# 

**Published:** 1853-10

**Authors:** 


### CONTENTS.

### ORIGINAL COMMUNICATIONS.

Answer to Dr. Drake's Interrogatories........1
A Birds-eye View of some of the More Important Points of Medico-Dental Practice, Illustrating the Importance of Thorough Education. By Dr. Wm. A. Pease........17
Clasps, by Dr. B. T. Whitney,........34
Practical Thoughts on Tooth Drawing. By C. T. Cushman, D. D. S.,........39
A few Suggestions to Young Men Commencing or about to Commence the Practice of Dentistry. By J. F. Johnston. D. D. S.,........45
Supernumerary Dens Sapientiæ. By J. Richardson, D. D. S.,........50

### CORRESPONDENCE.

Letter from Springfield, Ill.,........53

Letter from New Castle, Ky.,........56

### DENTAL ABSTRACTS.

Inflammation of Dentine or Tooth Bone,........57
Preparing the Mouth for full sets of Artificial Teeth,........60
Swaging Plates........60
Mortality of Children from first Dentition,........61
Dentistry in France,........62
Reproduction of the Inferior Maxillary Bone,........64
Replacement of the Teeth,........65
An Extraordinary Case of Hemorrhage,........65
Effects of Chloroform on the Blood,........67
Method of making Sets of Teeth with continuous Gums, as practiced by C.F. Delabarre,68

### EDITORIAL.

Prepared Gold for Filling Teeth,........72
American Journal of Dental Science,........72
Dental News Letter—Editorial Change,........72
Iowa Medical Journal,........73
Subscribers,........73
Teeth Swallowed,........73
The Present Number of the Register,........74
Thompson's Compound Self Adjusting Blow Pipe,........74
Our Advertising Department,........74
Dr. Bigelow,........74
Dr. Jacob A. Cook,........74

